# *GLRA1* mutation and long-term follow-up of the first hyperekplexia family

**DOI:** 10.1212/NXG.0000000000000259

**Published:** 2018-08-07

**Authors:** Martin Paucar, Josefine Waldthaler, Per Svenningsson

**Affiliations:** From the Section of Neurology, Department of Clinical Neuroscience, Karolinska Institutet, Stockholm, Sweden.

Hyperekplexia (HPX) is a rare familial disorder characterized by an exaggerated startle reflex and stiffness at birth. In 1958, Boris P. Silfverskiöld published a report on a Swedish family affected by “emotionally precipitated drop seizures.”^1^ This first description of HPX became seminal, but it would take 35 years before mutations in the glycine receptor subunit alpha-1 (*GLRA1*) gene were discovered as the cause of this disease.^2^ Subsequently, *SLC6A5* and *GLRB* mutations were discovered as causes of HPX.^3^ Here, we present a 60-year follow-up of the Silfverskiöld family found to harbor the R271Q mutation in the *GLRA1* gene. Some affected patients in this family display unreported features for HPX.

## Methods

This report was made within the frame of a study approved by the local ethics committee (*Etikprövningsnämnden* 2016/1661-31). The family consisted of 4 affected patients (figure). Phenotype details are provided in the original article and summarized in table e-1 (links.lww.com/NXG/A64). Briefly, 3 patients had early-onset violent and injurious falls triggered by unexpected stimuli (II-1, II-2, and III-1) causing a skull fracture in 2 (II-1 and II-2). Three patients had hypnagogic myoclonus and a good response to phenobarbital. At times, symptoms receded spontaneously in patient II-1. Reported onset in I-1 (J.E.) was at age 40 years; he had startle with falls once or twice per year. Siblings II-1 (A.W.) and II-2 (B.E.) lost consciousness sometimes after startle-related falls.^1^ Patient II-1 was diagnosed with late-onset dementia and died at age 87 years; patient II-2 was found drowned in a bathtub at age 76 years. Patient III-1 was aged 10 years at the time of the publication; at present, she is a 66-year-old retired school teacher. She was diagnosed with stiff baby syndrome. Patient III-1 had symptoms until age 13 years. At that point, symptoms receded spontaneously until age 27 years. When her symptoms reappeared, beneficial treatment with clonazepam was started and continued since then. Patient IV-1, born in 1976, presented with insidious clumsiness starting in childhood and later startle reactions. She underwent surgery for umbilical hernia at age 1 year. Stiffness became gradually persistent between startle reactions during adolescence; treatment with clonazepam was started at age 22 years. She has also been on continuous antidepressant treatment; Gabapentin was added later because of diffuse pain. She works part time as a preschool teacher. At age 32 years, she developed anxiety, weight loss, and gait difficulties. On examination, an exaggerated head-retraction reflex, hesitant gait, tremulous jerks in all extremities, and tremor of variable frequency were evident; the latter indicates a functional overlay. EMG displayed 80 ms polymorph bursts with a constant frequency of 7 Hz; there was no evidence of neuropathy; MRI of her brain was normal. The heterozygous mutation c.896G>A (R271Q) in *GLRA1* was found in patients III-1 and IV-1.

**Figure:**
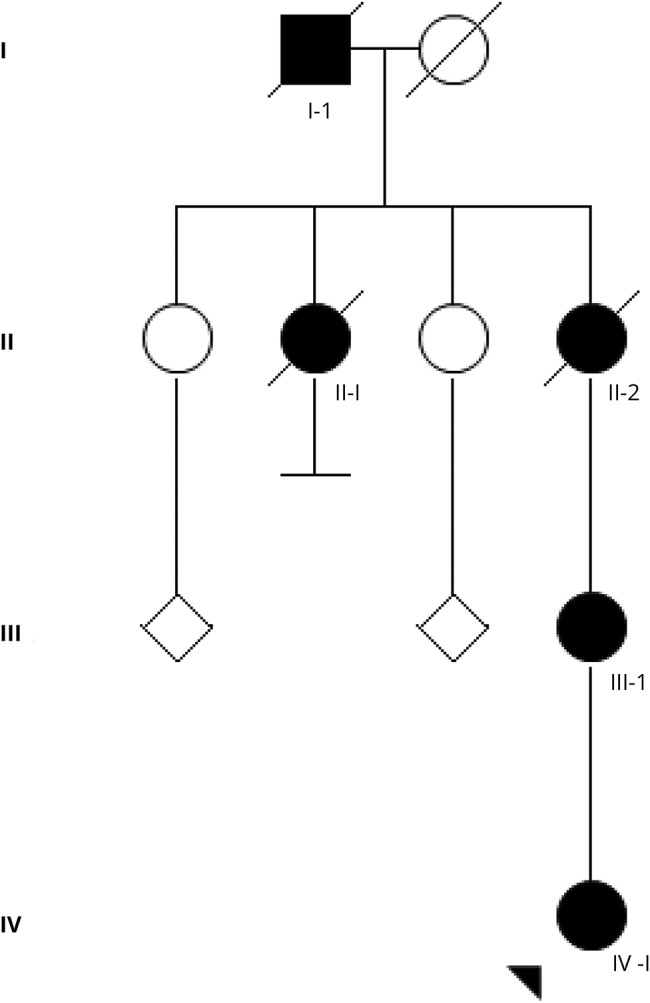
Updated pedigree of the Silfverskiöld hyperekplexia family described originally in 1958 Phenotype details on patients III-1 and IV-1 are provided in the text, both patients harbor the recurrent R271Q mutation in *GLRA1*.

## Discussion

The R271Q mutation in *GLRA1* found in the Silfverskiöld family is consistent with a phenotype previously described in association with *GLAR1* mutations. HPX phenotype was variable in this family, but there are some novel features. For instance, the waxing and waning course (patient II-1) and adult onset (I-1) have not been described in other *GLRA1* mutations. In addition, another patient (III-1) had a spontaneous remission for 14 years. Anxiety is unreported in HPX; in the index case, this feature could be related to fears of fall. Also unusual is the absence of perinatal stiffness, considered as one of the diagnostic criteria for HPX, and the functional overlay in the index case. Incongruence and variable frequency of tremor in the index case are compatible with a psychogenic movement disorder. Reports on the occurrence of psychogenic movement disorders in the context of a positive family history of hyperkinesias are scarce.^4^ HPX is responsive to clonazepam, and the Vigevano maneuver is effective during the neonatal period.^3^ HPX associated with *GLRA1* mutations is in most cases an autosomal recessive disease.^2,3^ Hypnagogic myoclonus and umbilical hernia have been described in association with *GLRA1* mutations. We did not find any of the other features associated with HPX such as apnea, seizures, developmental delay, learning disabilities, or sudden death.^3^ These features are more likely to occur in patients with biallelic *GLRA1* mutations or in HPX associated with mutations in *SLC6A5* and *GLRB*.^3^ Less often are heterozygous *GLAR1* mutations associated with mild developmental delay.^3^ R271Q is the most common dominant *GLRA1* mutation identified in families of different ethnicities; a founder effect among Caucasian patients has been proposed.^3,5^ R271Q is located in the second membrane-spanning domain of the receptor, which is expressed on the cell surface but displays reduced current and channel opening.^6^

HPX occurs also as part of severe neurodevelopmental syndromes associated with mutations in *ARHGEF9* and *GPHN*, the latter is a lethal condition, which illustrates the importance of etiologic diagnosis. A variety of animal models and in vitro studies have provided valuable knowledge on HPX associated with *GLRA1* mutations,^3,6,7^ but questions about pathophysiology and variable expressivity still await answers.
